# Immunomodulation as a Novel Strategy for Prevention and Treatment of *Bordetella* spp. Infections

**DOI:** 10.3389/fimmu.2019.02869

**Published:** 2019-12-13

**Authors:** Monica C. Gestal, Hannah M. Johnson, Eric T. Harvill

**Affiliations:** Department of Infectious Diseases, College of Veterinary Sciences, University of Georgia, Athens, GA, United States

**Keywords:** immunomodulation, *Bordetella*, Toll-receptors, adjuvants, pertussis

## Abstract

Well-adapted pathogens have evolved to survive the many challenges of a robust immune response. Defending against all host antimicrobials simultaneously would be exceedingly difficult, if not impossible, so many co-evolved organisms utilize immunomodulatory tools to subvert, distract, and/or evade the host immune response. *Bordetella* spp. present many examples of the diversity of immunomodulators and an exceptional experimental system in which to study them. Recent advances in this experimental system suggest strategies for interventions that tweak immunity to disrupt bacterial immunomodulation, engaging more effective host immunity to better prevent and treat infections. Here we review advances in the understanding of respiratory pathogens, with special focus on *Bordetella* spp., and prospects for the use of immune-stimulatory interventions in the prevention and treatment of infection.

**Graphical Abstract:**
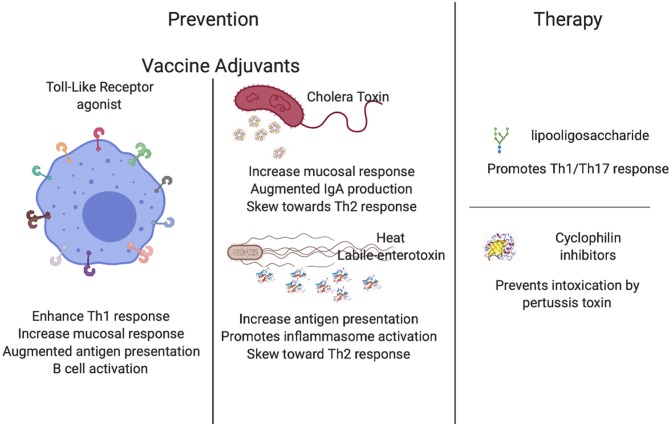
Areas of investigation focused on the use of immunomodulation in prevention and therapy of infectious diseases. Created with BioRender.

## Introduction to the Strategy of Immunomodulation for Health

We are exposed to vast numbers of pathogens during our lifespan, but only a small number manage to cause disease. Invading bacteria face a hostile environment in hosts with arrays of antimicrobial compounds and components of immunity. To persist in such an environment, bacteria must find a way to survive this onslaught of antibacterials. The strategy of resisting them all may be exceedingly challenging or impossible; instead, most of the best-studied pathogens have mechanisms that allow them to evade the full effects of host defenses (1–12). In this review, we will consider examples of novel approaches in vaccine and therapeutic development that have been guided by the better understanding of bacterial immunomodulatory abilities. We will focus on findings with *Bordetella* spp., considering novel adjuvants that enhance host immune response and new immunostimulatory therapies that can augment the most effective aspects of the host immune response. The results highlighted in this review demonstrate that the manipulation and/or disruption of bacterial immunomodulatory properties are providing a highly promising approach that could replace antibiotics in a near future. Understanding the mechanisms that bacteria utilize to manipulate host immune response, as well as the immune signaling pathways that lead to greater protective immunity, can guide the development of targeted interventions that can enhance the host immune response to more effectively kill the bacterial hazard.

## The Bordetellae; Biology; and Experimental System

Pertussis disease is caused by *B. pertussis*, a highly transmissible human pathogen that causes a respiratory illness also known as the 100-day cough (13). Among the proposed reasons for its resurgence are waning immunity (13), the end of the “honeymoon period” (14), the past vaccination calendar (15), and the failure of the current acellular vaccine to confer sterilizing immunity and long-lasting herd immunity. The increase in the number of cases is associated with more advanced diagnostic tools than ever before, allowing for an increase in the number of identified cases (16–31), but also with increased morbidity and mortality that creates an unambiguous imperative for improved prevention methods.

Vaccination has greatly increased life expectancy by preventing several historically notorious infectious diseases (32–36). However, we are witnessing a rise in several preventable diseases previously thought to be controlled (37), such as pertussis (38). Around 1945, a whole-cell vaccine against *B. pertussis* was introduced, causing an unprecedented decrease in the number of reported pertussis cases. However, due to undesirable adverse effects such as fever, erythema, swelling, drowsiness and others, this was replaced in several industrialized countries by an acellular vaccine that contains between 3 to 5 bacterial proteins (39–44). Despite the fact that both types of vaccines generate antibodies that impede bacterial adhesion and have bactericidal action, these have not been sufficient to halt the increase in the number of cases. In response to this increase a boost was introduced to extend immunological memory, and new vaccination strategies targeted to pregnant women and close family have also been introduced as an attempt to protect highly susceptible newborns (45–48).

As the number of cases continues to increase, the scientific community is working to understand the causes that drive this reemergence (13, 49). Amongst the proposed causes of this increase are, limitation to the protection conferred by the current acellular vaccine. Not only does the acquired anamnestic response wane rapidly (50), but the acellular vaccine still allows for bacterial colonization of the nasal cavity and shedding. Combined, these factors illuminate the fact that the current vaccines used in most industrialized countries still permit transmission of pertussis from host to host (51–54), which has even more significant impacts when considered in tandem with the rise of anti-vaccination movements. Yet another cause for the increase is the differences detected in the immune response triggered by the whole cell vaccine (Th17) vs. the acellular vaccine (Th2) (51, 55–57). It is important to highlight that while neither whole-cell nor acellular vaccines confer long-lasting immunity, and the merits of both responses have been debated in recent years, the general consensus agrees on advantages to skewing T cell response toward Th1/Th17 immunity (58–61).

The “gold standard” of immunity to pertussis is considered to be the classical Th1/Th17 T cell response induced by convalescent immunity (62); however, there is significant cumulative evidence that infection-induced immunity is imperfect and shorter-lived than it could be (50). Current discoveries contribute to better understanding of the immune response to Bordetellae, and the important role that CD4 resident T cells play in a local memory response has been recently demonstrated (63). Another hypothesis is that Bordetellae are evolving, and due to the genome plasticity and adaptability of this pathogen, current isolates of *B. pertussis* have lost some of the antigens included in the acellular vaccine. This phenomenon is referred to as “vaccine driven evolution,” which helps justify why immunity is not as robust as it has previously been (64–67).

These are only some of the potential causes that are currently being considered, and it is most likely an uneven combination of all of them that is truly driving this pertussis resurgence. Although the whole-cell vaccine is still used, the trend is shifting toward a safer acellular vaccine, and efforts on improving their performance and the length of protective memory these generate will be discussed in this manuscript.

The current strategy for the development of vaccines is driven by the hypothesis that antibodies provide strong protection. As a consequence, most of the current acellular vaccines are highly safe and generate a rapid antibody response that is protective, albeit limited (68, 69). Importantly, infection triggers a complex and well-orchestrated sequence of responses involving many interacting components of innate and adaptive immunity, directed by several signaling pathways that present numerable known, and probably many more unknown, opportunities to interfere in the succession of events that can skew the resulting immune response.

Bordetellae harbor multiple mechanisms that allow them to modulate the host immune response (1, 70, 71). Some of the proteins that these organisms utilize to manipulate immune cells include adenylate cyclase toxin (ACT), a pore forming protein that leads to the deregulation of cAMP levels within target cells (72, 73); type 3 secretion system (T3SS), a needle-like structure that injects toxins within mammalian cells (74–76); a type 6 secretion system that uses a phage-like mechanism to inject molecules (77); pertussis toxin (PTX), which prevents G proteins from interacting with G protein-coupled receptors on the cell membrane and therefore interfering with intracellular communication (78–80); and filamentous hemagglutinin (FHA), which binds signaling receptors, enables adhesion to epithelial cells and interferes with cytokine production (81, 82). Based on these studies of various immunomodulators we can now begin to adjust the way we design preventative and responsive medications to fight bacterial infections in more effective ways.

A good understanding of the sequential reactions of the immune response (and bacterial manipulation of them) is key to improving the induction and maintenance of robust long-lasting protective immunity. Some of the *Bordetella* spp. virulence factors are already being investigated for treatments, such as PTX for human immunodeficiency virus (HIV) treatment (83–89). Understanding how we can alter bacterial ability to sense and respond to the host to modulate its response can lead to treatments and therapies that focus on the enhancement of more appropriate and effective host immune responses.

## Immunotherapy in Prevention

### Adjuvants

The *Bordetella pertussis* acellular vaccine has not completely blocked the spread of pertussis because it allows for colonization of the nasal cavity (48) and provides only temporary protection (13). Adjuvants are well-documented for their potential to increase vaccine performance, and some adjuvants such as CpG oligodeoxynuceotides or alum are commonly found in vaccine formulations (90, 91). There are a plethora of adjuvants that can potentiate the performance of a vaccine and can be classified into two main groups: Toll-like receptors agonists (92–94) and mucosal adjuvants (58, 95–97). These two distinct classes have been closely considered for their contributions to pertussis vaccines as well as therapeutics (98–103), yielding highly promising enhancing properties.

### Toll-Like Receptors Agonists

Toll-Like Receptors (TLRs) are highly sophisticated sentinels that recognize specific pathogen-associated molecular patterns (PAMPs). The differential activation of TLRs is one of the main determinants for an efficient immune response against pathogens. Under this premise, researchers have been working on the addition of TLR agonists to vaccines with the expectation that activating different TLRs will command the type of T cell response produced (104) and will ultimately enhance host protective immunity (105).

One of the best studied Toll-Like Receptors is TLR2, which recognizes a broad spectrum of bacterial cell wall components, including lipopolypeptides, peptidoglycan, and lipochoic acids, that trigger different signals that shape the immune response against the bacterial threat (106). It has been demonstrated that the use of TLR2 agonists as adjuvants to already developed vaccines increases immunity, especially in neonates (93). This feature is highly relevant to the design of vaccines against diseases that primarily affect newborns and young infants (93). Moreover, TLR2 agonists in combination with the BCG vaccine can enhance protection against *Mycobacterium tuberculosis* (107), skewing the cellular response toward Th1 (100), and resulting in a more robust protective memory response, further promoting its use in vaccinology. TLR2 has been also correlated with an efficient response to *B. pertussis* infections (108), and some preliminary data has revealed that the use of these agonists enhances protection against infection by pertussis (58, 100). Altogether, these data suggest that TLR2 agonists may be promising candidates to combine with current or new vaccines to enhance the protective response.

Similarly, TLR4 appears a good candidate for vaccine enhancement because it recognizes lipopolysaccharide (LPS) molecules, which are commonly present on the surface of most bacteria. Agonists of TLR4 enhance the performance of several vaccines including viral, bacterial, and even mycobacterial (109–113). One important aspect is its promotion of mucosal immunity (114–116), which is critical for the generation of protection against certain infections including gut and respiratory diseases like pertussis (117–119), although this increase is achieved via mucosal delivery of the vaccine rather than systemic (120). Molecular evidence has revealed that the addition of a TLR4 ligand to the acellular pertussis vaccine resulted in a shift from a Th2-dominant response to additional induction of Th17 (121, 122). The abundant immunological evidence that highlights the role of TLR4 in the immune response to *B. pertussis* (102, 123–130) indicates that TLR4 agonists are promising candidate for the generation of more robust protective immunity.

TLR5 (131) is also a highly plausible candidate to augment vaccine performance since it recognizes flagella, which are present in a multitude of bacterial species. Previous literature has indicated that ectopic expression of flagella in *Bordetella* spp. leads to faster clearance of the infection (132), and it was later revealed that TLR5 activates antigen-presenting cells, increasing T cell response (133) (*manuscript in preparation*), and may ultimately contribute to the more rapid clearance previously reported. In several other microorganisms, the addition of TLR5 agonists have resulted in an increased performance of the vaccine (134–141). Altogether these data suggest that TLR5 agonists could significantly increase the performance of the current acellular pertussis vaccine.

TLR7 recognizes single-stranded RNA (142–153) and has been demonstrated to be a promising vaccine adjuvant for protection against several microorganisms (154, 155). Similar to TLR2, the TLR7 agonist augments immunity in newborns, the most susceptible population (93, 102, 143, 156, 157). The addition of a TLR7 agonist to an alum-adjuvant of pertussis vaccine skewed the immune response toward Th1/Th17 and significantly decreased colonization (98), providing preliminary data to further pursue this agonist in other animal models.

Lastly, TLR9 recognizes unmethylated CpG oligodeoxynucleotides and promotes IL-6 secretion and consequent B cell activation (158–168). It has been demonstrated that enhancement of TLR9 receptors augment activity of antigen-presenting cells in neonates (93, 102, 169). Addition of a TLR9 agonist to the acellular pertussis vaccine resulted in greater stimulation of B and T cells and a shift to Th1, as well as higher antibody titers (81, 170–174), suggesting that an agonist of TLR9 is also a candidate to add to the current pertussis vaccines. These have the potential to be widely used agonists, as most of the current vaccine's efficacy is measured as an increase in antibody titers.

Altogether, these results demonstrate that TLR agonists are great candidates to be used as vaccine adjuvants to increase protective immunity. Interestingly, some of the TLR agonists substantially augment vaccine performance in newborns and infants, which represent the most susceptible population (93, 169) although there are substantial hurdles to applying this knowledge. Moreover, preliminary data obtained with TLR2, and TLR7 agonists demonstrate the improved performance of the current *B. pertussis* vaccine and indicates that the use of adjuvants can feasibly potentiate and augment the generation of protective immunity (58, 98, 100).

### Mucosal Adjuvants

Adjuvants have been used to potentiate, enhance, or accelerate vaccine effects since the 1920s (105) and the field has greatly evolved since. Mucosal adjuvants include cholera toxin, heat-labile enterotoxin, and other compounds have been studied for their particular ability to increase protection on mucosal surfaces (175). These are of extreme importance, not only because of the aforementioned increase in vaccine performance, but also because the delivery method involving intranasal vaccination has a lot of potential for improving the delivery of the vaccine and increasing acceptance among needle-phobic population. In the following paragraphs we will detail the mechanisms of action and the data compiled for some of the most promising mucosal adjuvants.

Cholera toxin (CT) and heat-labile enterotoxin of *Escherichia coli* (LT) are highly antigenic; however, due to their toxicity, they are not ideal candidates for human therapies. Recently, safe forms of these toxins created via genetic manipulation have been utilized as adjuvants to enhance the function of mucosal vaccines (103, 176–181). The mechanism behind this augmented immune response induced by CT is an increase in the permeability of the mucosal epithelium, enhanced antigen presentation, the consequent promotion of dendritic cell maturation, increased IgA response, and finally, the generation of complex stimulatory and inhibitory effects on T cell proliferation and cytokine production such as IL-4, IL-5, IL-6, and IL-10 that skew the response toward a Th2-type (177, 182). CTA1 is the subunit responsible for the immunomodulatory activity in conjunction with ERdj5 in the endoplasmic reticulum, which is the target for CT. In the absence of ERdj5, mice failed to produce inflammatory cytokines, indicating that CT action requires ERdj5 (183). Similarly, the calcium-binding protein S100A4 is required for efficient antigen presentation and enhanced activity of CT, as it is necessary for the humoral and cellular response (184). CT has been tested as an adjuvant for pertussis vaccine and preliminary data suggests that it substantially improves mucosal protection by augmenting IgA levels (183, 185), and it has even been suggested that this adjuvant may be safe for use in humans (186, 187). Some studies have revealed that conjugation of CT with pertussis toxoid added to the current acellular vaccine (188) or Fimbriae (Fim2) (189) are highly promising candidates to improve the generation of protective immunity from these vaccines.

Similar to CT, the heat-labile enterotoxin from *E. coli* (LT) promotes an antigen-specific response inducing IgA antibodies, Th17 response, and the enhancement of long-lasting protective immunity (190) while also being safe for use in humans (191). LT promotes maturation of dendritic cells, antigen-specific IL-17 positive cells, and production of IL-1α, IL-1β, and IL-23 by dendritic cells. Trials in animals have revealed the efficacy of this adjuvant at enhancing mucosal response (192). LT promotes dendritic cell maturation enhancing IL-1β production through activation of caspase-1 and the NLRP3 inflammasome complex. Simultaneously, LT enhances LPS-induced IL-1α and IL-23 expression through activation of ERK MAPK in dendritic cells inducing the development of Th17 T cells (193). Interestingly, LT derivatives LTK63 (non-toxic mutant of LT) and LTR72 (which retains partial enzymatic activity) revealed two distinct phenotypes characterized by stimulation of IL-12 and TNF-α production by macrophages, resulting in enhanced Th1 responses with the LTK63 adjuvants. In contrast, LTR72 suppresses LPS-induced IL-12 production, increases type 2 responses, inhibits Th1 response, and facilitates clearance of bacterial burden (194), demonstrating that both subunits of the toxin have particular activities that can be beneficial for the improvement of the current acellular pertussis vaccine.

Another mucosal adjuvant that is widely investigated is retinoic acid, a powerful immunomodulator that interferes with growth, differentiation, and other aspects of the cell life cycle. Importantly, retinoic acid is also essential in the generation of mucosal immunity, the promotion of tolerogenic effects, the generation of a robust innate and adaptive immune response, and moreover, it also acts as a negative regulator of IgE production (195–197). It has been hypothesized that retinoic acid plays a fundamental role in sustaining mucosal homeostasis by down-regulating IgE levels (197). Its performance as an adjuvant has been studied in several organisms and the plethora of results obtained have revealed that retinoic acid is a promising candidate to use as an adjuvant of mucosal vaccines by itself or encapsulated in nanoparticles (198–203). Unfortunately, its activity in conjunction with the pertussis vaccine has not yet been assessed.

The use of biopolymers in mucosally-administered vaccines has substantially improved the current vaccine formulations and has great potential for the future (204). Some of the presently investigated biopolymers include alginate (205–212) and gellan (213, 214). Although these are still in early stages of study, other biopolymers, such as chitosan (95–97, 215–232), starch (233), and β-glucan (234–241), have already been tested in animal trials with encouraging success. While the use of biopolymers is still rising, this area of investigation is highly promising, especially for enhancement of mucosal protection. Mucosal delivery has been explored for pertussis immunization from different approaches that have resulted in hopeful results in which Th17 response was enhanced and the animals were more robustly protected against challenge (58, 170, 242, 243).

To summarize, several mucosal adjuvants are being investigated, some of which are derived from toxins while still others are derived from biopolymers. Both act to enhance the performance of vaccines, particularly those that can be orally or intranasally delivered, usually in cases in which mucosal protection is a key component of immunity. However, these further demonstrate that different strategies and approaches can be used to improve the performance of the current vaccines to produce and enhance individual and herd immunity.

### Novel Vaccination Strategies

The combination of BCG and acellular pertussis vaccination has been shown to reduce the mortality rate of pertussis (244–247). Immunological studies unraveling the underlying mechanism by which protection against pertussis is enhanced are necessary. Some groups have focused on the addition of antigens to the current vaccine in order to improve performance. After demonstrating via *in vitro* experiments that the autotransporter BrkA would be a good candidate to generate antibodies that kill *Bordetella* spp., BrkA has been tested as an adjuvant of the current acellular pertussis vaccine, the results of which revealed robust lung protection against infection with *B. pertussis* (248, 249). Two other autotransporters, Vag8 (250, 251) and SphB1, when added to the current pertussis vaccine resulted in improved protection against *B. pertussis* infection (252). Adenylate cyclase toxin (ACT), when added to a current vaccine formulation significantly decreased inflammation and increased the generation of protective immunity (253, 254). BcfA (colonization factor A) has been used as adjuvant in the current vaccine, and the preliminary data obtained with the murine model reveals that the addition of this adjuvant shifts the T cell response toward Th1/Th17 (255).

Live vaccines have the potential to induce strong mucosal protection, but suffer from concern about their risk. An exciting new vaccine candidate against *B. pertussis* is the live attenuated vaccine, BPZE1, which has been shown to induce a robust local B and T cell response (256–282) despite genetically engineered mutations that render it relatively safe (283, 284). Excitingly, phase I trials demonstrate that the intranasal formulation of the vaccine transiently colonizes the nasal cavity, leading to the generation of stronger immunity (264, 268).

Several groups are currently working on the development of outer membrane vesicles and outer membrane proteins in protection against *B. pertussis* as well as cross-protection against several *Bordetella* spp. and characterizing the immune response as well as protective immunity (285–295). In animal studies, immunization with outer membrane vesicles led to not only better humoral and cellular (Th17) memory, but also to a significant increase in IgA titers, which is one of the major hurdles of current vaccination strategies against this pathogen (296–298). It is important to highlight that the increase in IgA responses upon immunization with outer membrane vesicles is only obtained when these are administrated mucosally (299). The classic delivery for OMV's, which is subcutaneous or intraperitoneal immunization, does not induce IgA responses and this novel delivery method provides a great advance, as it can be administered with more ease and induces an even better immunological response. The increase in mucosal protection has led to efforts toward improved nasal delivery approaches and a thermostable spray containing outer membrane vesicles has been developed. This spray significantly improves delivery and decreases the discomfort other intranasal formulations might cause. Importantly, this delivery method still maintains all the outstanding qualities of the classical delivery of these purified outer membrane vesicles (300).

Finally, another highly promising strategy is focused on the disruption of bacterial ability to manipulate the host immune response. Under the premise that bacteria harbor mechanisms that allow them to sense and respond to host immunity, disrupting these pathways would allow for the generation of more robust protective immunity. A live attenuated vaccine in which immunomodulatory mechanisms are disrupted might confer cross-protection against classical Bordetellae, which are known to share many antigens. Although this is only the first study for this method of vaccine design (*manuscript in revision*), this novel approach has great potential for the generation of new vaccine candidates and possibly therapeutics.

## Immunotherapy in Treatment

### LOS-Derived Oligosaccharide Glycoconjugates

Pertussis toxin (PTX) in an inactivated form (PTd) functions as a major protective antigen, stimulating production of toxin-neutralizing antibodies which can protect against damage caused by the toxin, but do not target the bacteria itself (301, 302); however, it also demonstrates possible partial reversion back to its toxic active form (303, 304), which may be responsible for the reactogenicity seen in a small percentage of vaccine recipients. It is also a secretory protein, which is only loosely associated with the cell and is therefore not an ideal target for bactericidal antibodies. A more effective target is an abundant surface component such as the endotoxin lipooligosaccharide (LOS), an LPS analog with a complete absence of the O-specific polysaccharide chain that is produced by several varieties of Gram-negative bacteria (305). LOS provides significant adjuvant properties via induction of IL-12 and 1L-1β that promote Th1 and Th17 responses, respectively (306, 307). It also displays pyrogenic, mitogenic, and endotoxic activity that necessitate its conjugation or conversion to a less destructive form prior to its use in a vaccine.

LOS conjugated to protein carriers filamentous hemagglutinin, bovine serum albumin, and tetanus toxoid (TTd) successfully induce a strong bactericidal response specific to LOS presented on the surface of *B. pertussis*, leading to complement-mediated destruction of the cell (90, 308, 309). These protein carriers are also surface components, like LOS, and the resulting surface-associated conjugate acts as a strong target for antibody action directed against *B. pertussis*.

Somewhat surprisingly, another conjugate iteration in which an LOS-derived oligosaccharide is covalently linked with the secretory protein PTX yields a uniquely non-toxic and immunogenic glycoconjugate that retains the antigenic properties of PTX while also inducing the production of bactericidal antibodies. The presumed linkage at the fetuin- and glycoprotein-binding sites of PTX inactivates the enzymatic activity of the protomer A and binding properties of oligomer B, demonstrated using *in vitro* assays (310). Although the use of LOS appears to be highly promising, *in vivo* studies still need to be done to assess pharmacological parameters of safety and biodistribution.

### Cyclophilin Inhibitors

PTX is internalized in cells via endocytosis and then follows a retrograde transport system to the endoplasmic reticulum. The enzymatically active (A) subunit of PTX, PTS1, detaches from the rest of the toxin in the ER and unfolds due to its thermal instability. It is then transported into the cytosol with the help of cyclophilin (Cyps), an important protein folding helper enzyme that also is required to facilitate membrane translocation from early endosomes into the cytosol of various ADP-ribosylating toxins (311–313). Inhibiting Cyps activity has been shown to in turn inhibit membrane translocation and protect cells from intoxication with PTX and others (311).

Inhibition can be achieved via the approved immunosuppressive drug cyclosporine A (CsA), which specifically inhibits Cyps activity in mammalian cells by binding directly to Cyps and forming a ternary complex. It has been used as the primary agent in immunosuppressive regimens such as grafts and transplants since the 1980s. It is now suggested that CsA might interfere with the translocation of PTS1 from the ER into the cytosol; it may also play a role in reassembling the unfolded PTS1 subunit (311).

*In vitro* intoxication assays performed on CHO-K1 cells demonstrated that CsA-treated cells were protected from PTX intoxication. Interestingly, up to 50% of CsA is retained intracellularly, even in the absence of extracellular inhibitor, after 18 h (314). Thus, presumably, intracellular Cyps stay inhibited over a longer period of time, explaining the toxin-resistant phenotype. This is also concomitant with the long retention of CsA in different tissues observed after CsA administration in human patients (315, 316). This inhibitor was delivered orally during trials, but its use in a mucosal spray or as a directly injectable vaccine component has yet to be investigated.

## Future Directions and Conclusion

Since the years of our notoriously premature celebration of victory over infectious disease, there has been seemingly inexorable retaliation. There is now justifiable concern, shifting toward fear, about the combined threats of increasing antibiotic resistance and the failures of current vaccines due to factors including incomplete vaccine uptake, vaccine-driven evolution and other threats. However, recent advances in our understanding of immunology and the tools to manipulate it present hope for more rational targeted interventions that are focused on enhancing the natural host response. Similarly, improved understanding of strategies and mechanisms by which bacteria modulate the immune response provides new targets for treatment and prevention. In the coming years, we will likely witness an expansion in the field of immunotherapy promoted by a better understanding of the finely tuned interactions of bacteria and host.

## Author Contributions

MG: original idea of the manuscript, writing, and editing. HJ: writing and editing. EH: editing and final approval.

### Conflict of Interest

The authors declare that the research was conducted in the absence of any commercial or financial relationships that could be construed as a potential conflict of interest.
